# Observing femtosecond orbital dynamics in ultrafast Ge melting with time-resolved resonant X-ray scattering

**DOI:** 10.1107/S2052252523007935

**Published:** 2023-09-30

**Authors:** Heemin Lee, Je Young Ahn, Sae Hwan Chun, Do Hyung Cho, Daeho Sung, Chulho Jung, Jaeyong Shin, Junha Hwang, Sung Soo Ha, Hoyoung Jang, Byeong-Gwan Cho, Sunam Kim, Jaeku Park, Daewoong Nam, Intae Eom, Ji Hoon Shim, Do Young Noh, Yungok Ihm, Changyong Song

**Affiliations:** a Department of Physics, Pohang University of Science and Technology, Pohang 37673, Republic of Korea; b Center for Ultrafast Science in Quantum Matter, Max Planck POSTECH/Korea Research Initiative, Pohang 37673, Republic of Korea; c Photon Science Center, Pohang University of Science and Technology, Pohang 37673, Republic of Korea; d Department of Chemistry, Pohang University of Science and Technology, Pohang 37673, Republic of Korea; e Pohang Accelerator Laboratory, Pohang 37673, Republic of Korea; f Department of Physics and Photon Science, Gwangju Institute of Science and Technology, Gwangju 61005, Republic of Korea; g Institute for Basic Science, Daejeon 34126, Republic of Korea; Harima Institute, Japan

**Keywords:** ultrafast melting, resonant X-ray scattering, X-ray free-electron lasers

## Abstract

Photoinduced nonequilibrium phase transitions have stimulated interest in the dynamic interactions between electrons and crystalline ions, which have long been overlooked within the Born–Oppenheimer approximation. Ultrafast melting before lattice thermalization prompted researchers to revisit this issue to understand ultrafast photoinduced weakening of the crystal bonding. However, the absence of direct evidence demonstrating the role of orbital dynamics in lattice disorder leaves it elusive. By performing time-resolved resonant X-ray scattering with an X-ray free-electron laser, we directly monitored the ultrafast dynamics of bonding orbitals of Ge to drive photoinduced melting. Increased photoexcitation of bonding electrons amplifies the orbital disturbance to expedite the lattice disorder approaching the sub-picosecond scale of the nonthermal regime. The lattice disorder time shows strong nonlinear dependence on the laser fluence with a crossover behavior from thermal-driven to nonthermal-dominant kinetics, which is also verified by *ab initio* and two-temperature molecular dynamics simulations. This study elucidates the impact of bonding orbitals on lattice stability with a unifying interpretation on photoinduced melting.

## Abstract 2

Bonding orbital dynamics in ultrafast melting have been directly observed using femtosecond time-resolved resonant X-ray scattering at the Pohang Accelerator Laboratory X-ray Free-Electron Laser, and smoking-gun evidence on direct linkage between bonding orbitals and lattice stability unifying thermal-to-nonthermal reactions to explicate photo-induced phase transitions is provided.

## 1. Introduction

Networked spatial arrangements of ions, bound tightly by shared valence electrons, become fundamental building blocks of crystals that host various physical properties to include topologically protected extraordinary dynamics of charges and spins, superconductivity with cooperative dynamics of electrons and the lattice *etc*. As such, lattice stability plays an essential role in inducing emergent physical properties with exotic phases to facilitate ongoing research activities (Born & Huang, 1954 ▸; Ichikawa *et al.*, 2011 ▸; Beaud *et al.*, 2014 ▸; Stojchevska *et al.*, 2014 ▸; Buzzi *et al.*, 2018 ▸). Lattice energetics are intertwined with various interactions, including ionic Coulomb interactions and electron correlations, which complicate the investigation of structure-based phase changes. Femtosecond (fs) infrared (IR) laser pulses enable us to access electron-triggered phase transitions by promoting active research on photoinduced ultrafast phenomena (Nasu, 2004 ▸; Gamaly, 2011 ▸; Mathias *et al.*, 2012 ▸; Buzzi *et al.*, 2018 ▸; Ihm *et al.*, 2019 ▸).

In particular, the nonthermal melting that displays lattice disorder before ion thermalization has attracted special interest as a fundamental science issue concerning the simplest yet essential building blocks of solids (Siders *et al.*, 1999 ▸; Rousse *et al.*, 2001 ▸). The critical role of bonding orbitals in maintaining a crystal lattice becomes reappreciated by observing lattice disorder from photo-depleted bonding charges without disturbing ions. Femtosecond photoexcitation of bonding electrons weakens crystalline bonding, and even abruptly breaks the lattice order by changing the electronic potential energy surface (PES), as described by the bond-softening effect in covalently bonded crystals (Fritz *et al.*, 2007 ▸; Jung *et al.*, 2021 ▸). Also, by phase-matching ionic vibrations, coherent phonons can be induced to drive the ultrafast melting, as shown by the optical property measurements, X-ray diffraction (XRD) *etc*. (Pfeifer *et al.*, 1992 ▸; Zeiger *et al.*, 1992 ▸; Chatelain *et al.*, 2014 ▸; Zhu *et al.*, 2015 ▸). There have been various physical pictures that successfully account for ultrafast nonthermal lattice disorder, but experimental evidence directly verifying the photoinduced reconfigurations of the bonding electrons has been absent. This implies that the first physical process of photoexcited electrons to disturb the crystal bonding has been veiled, or that underlying physical mechanisms at the fundamental level describing the impact of bonding orbitals remain elusive (Siders *et al.*, 1999 ▸; Gamaly, 2011 ▸; Medvedev *et al.*, 2015 ▸; Lian *et al.*, 2016 ▸).

## 2. Experimental

**Figure 1:**
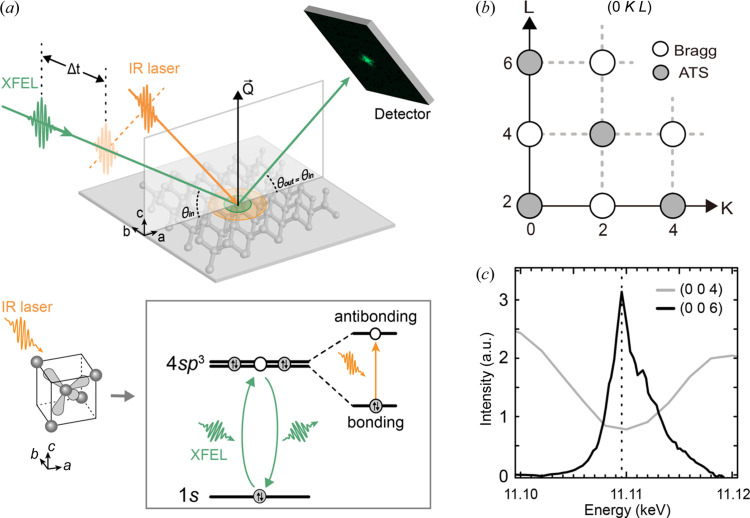
Bonding orbital dynamics of Ge probed by time-resolved resonant X-ray scattering. (*a*) Ultrafast orbital dynamics of Ge in relation to the lattice disorder is investigated by fs tr-RXS using XFEL pulses. Valence electrons are photo-excited from tetrahedral bonding to antibonding states by the fs-IR laser (orange arrows) to modify the 4*sp* ^3^ orbital configurations directly probed by Ge *K*-edge RXS. (*b*) Reciprocal space map (0 *K* *L*) shows Bragg reflections (open circles) and ATS (filled circles). (*c*) With the X-ray energy tuned to Ge *K*-edge (11.1095 keV), the sensitivity to 4*sp* ^3^ valence orbitals is enhanced to detect the forbidden ATS reflection.

To understand the femtosecond dynamics of electrons in bonding orbitals related to lattice disorder, we performed time-resolved resonant X-ray scattering (tr-RXS) experiments at the Pohang Accelerator Laboratory X-ray Free-Electron Laser (PAL-XFEL). An ultrafast time-resolved investigation was performed using femtosecond Ti-sapphire laser (λ = 800 nm) pumping and X-ray free-electron laser (XFEL) probing [Fig. 1 ▸(*a*), see Section S1 of the supporting information for details]. Temporal resolution was maintained at better than 200 fs (Kang *et al.*, 2017 ▸). Single-pulse XRD patterns were collected, and any unwanted data collection from damaged sample areas was prevented by moving the X-ray and IR laser illumination spots across the crystal (see Section S2 of the supporting information for details).

High-purity Ge crystals of the [001] direction cut wafers were used for the experiments. XRD patterns in specular reflection geometry (incident angle equal to the scattered angle) cover the (0 0 *L*) reflections. Here, *L* = 4*n* allows Bragg reflections of the crystal lattice for any integer value of *n*. The reflections at (0 0 4*n*+2) are systematically forbidden due to the glide plane symmetry of the Ge ions in the space group *Fd* 3 *m*. The extinction rule restricts the (0 0 4*n*+2) reflections to be forbidden even with anisotropic bonding or anharmonic thermal motion. They are distinguished from the observable (2*n* 2*n* 2*n*) reflections, even weak, due to aspherical charge distribution of the Ge ions; this is caused by charges distributed along the (111) directional covalent bonds (Roberto & Batterman, 1970 ▸). However, (0 0 4*n*+2) reflections are forbidden by the glide plane symmetry and can only be observed when the X-rays gain sensitivity to anisotropic orbital environments, known as anisotropic tensor susceptibility (ATS) scattering (Dmitrienko, 1983 ▸; Murakami *et al.*, 1998 ▸). This is realized for the resonant X-ray scattering (RXS) process by resonantly enhancing the X-ray sensitivity to a specific atomic orbital through orbital-specific electric multipole transitions (Finkelstein *et al.*, 1992 ▸; Templeton & Templeton, 1994 ▸; Murakami *et al.*, 1998 ▸; Ji *et al.*, 2003 ▸). We used the Ge *K*-edge to observe the 4*p* orbitals that directly mediate the covalent bonding of the Ge crystal via 4*sp* ^3^ hybridization [Figs. 1 ▸(*a*) and 1 ▸(*c*)]. As such, RXS intensity of the (0 0 4*n*+2) reflections will directly reflect orbital states involved in the covalent bonding of Ge (Elfimov *et al.*, 2001 ▸).

Photoinduced dynamics of the bonding orbitals related to the lattice disorder were investigated by monitoring (0 0 6) ATS and (0 0 4) Bragg reflections. We have chosen (0 0 4*n*+2) forbidden reflections with their exclusive sensitivity to the 4*sp* ^3^ atomic orbitals. These ATS reflections are resistant to extrinsic effects such as anharmonic lattice distortions, defects *etc*. and are responsive to intrinsic orbital configuration; the aforementioned (2*n* 2*n* 2*n*) reflections are susceptible to such charge related defects, thermal motions *etc*. (Tsai *et al.*, 2019 ▸). Tr-RXS directly monitors the dynamics of Ge bonding orbitals and ions disturbed by fs-IR lasers. IR photo-excitation redistributes electrons from bonding to antibonding orbitals by reducing the Ge bond strength [Fig. 1 ▸(*a*)] (Zürch *et al.*, 2017 ▸). By recalling that the RXS tensor is obtained as 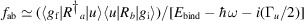 , one can know that the IR photoexcitation re-arranges the valence shell *u* to modify the intensity. Here, *g*_i(f)_ describes the initial (final) core-level state (*g*_f_ = *g*_i_ for elastic scattering in our work); *u* for the unoccupied valence shell with the energy bandwidth Γ_*u*_, *E*_bind_ = *E_u_* − *E*_g_ for the core–shell binding energy and ℏω for the incident X-ray energy. Tuning the X-ray energy to the *K*-edge of Ge promotes multipole transitions of electrons in the 1*s* core level to accommodate resonantly enhanced sensitivity to specific valence orbitals of corresponding transitions [Fig. 1 ▸(*c*)]. In electric multipole (2*^L^*) transitions, denoted E*L*, the *R* in the matrix element describes the electromagnetic interaction of the vector potential (***A***) for the X-rays in polarization (***ɛ***) with the electron momentum (***p***), ***A*** · ***p*** ≃ (***ɛ*** · ***p***)exp(*i* ***k*** · ***r***), for which dominant contributions are considered from the expansion of the vector potential [exp(*i* ***k*** · ***r***)] as ∼1 + *i* ***k*** · ***r*** for *E*1 (1*s*-to-4*p* transition for Ge *K*-edge) and *E*2 (1*s*-to-3*d*) in order (see Section S3 of the supporting information for details).

## 3. Results and discussion

**Figure 2:**
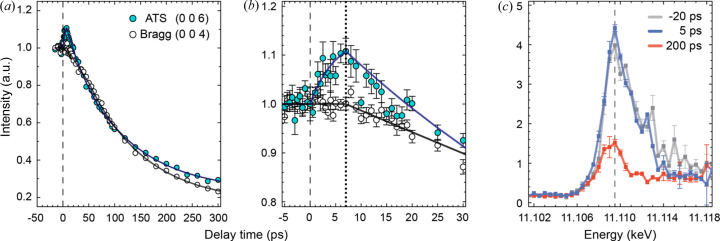
Temporal evolution of (0 0 6) orbital-sensitive and (0 0 4) Bragg reflections. (*a*) Diffraction intensities are compared for lattice (open circles) and orbital-sensitive reflections (filled circles) measured at an IR laser fluence of 45 mJ cm^−2^. Intensity decay is commonly noted for a delay time longer than ∼10 ps. (*b*) The time-dependent diffraction intensity is enlarged for early delay time. The intensity of the orbital-sensitive reflection related to the dynamics of valence orbitals increases immediately after the laser illumination, while the lattice reflection remained constant until ∼7 ps. (*c*) The resonance energy profiles of the orbital-sensitive (0 0 6) reflection are compared for various delay times displaying the same resonance feature. Decreased intensity at 200 ps resulted from thermalized disorder of the crystal lattice.

The intensity variation of the orbital-sensitive (0 0 6) ATS reflection was compared with that of the (0 0 4) Bragg reflection for the laser fluence at 45 mJ cm^−2^ (Fig. 2 ▸). Both Bragg and ATS intensities decreased for ∼300 ps due to lattice thermalization [Fig. 2 ▸(*a*)]. However, careful inspection revealed that the orbital-sensitive reflection displayed a distinct temporal evolution different from the lattice [Fig. 2 ▸(*b*)]. The intensity of the (0 0 4) reflection showing the lattice order remained intact until 7 ps, before it started to decrease. This lagged response showed delayed lattice thermalization requiring sufficient time for electrons to release excessive kinetic energy to ions via the electron–phonon scattering (Sundaram & Mazur, 2002 ▸).

However, as the most striking feature revealing the dynamics of bonding orbitals, the intensity of the orbital-sensitive (0 0 6) reflection changed promptly upon laser illumination. It increased monotonically to reach ∼11 % enhancement by 7 ps, then reversed the direction to decay concomitantly with the onset of lattice disorder [Fig. 2 ▸(*b*)]. The (0 0 6) ATS reflection, with direct sensitivity to valence orbitals, explicitly visualized the dynamics of orbitals involved in the crystal bonding as the essence of photoinduced ultrafast melting. Rapid response of this reflection results from the photoexcitation of the 4*sp* ^3^ bonding orbital electrons to the antibonding and nonbonding orbitals. The energy profile of the (0 0 6) reflection sustains its resonance character even after laser illumination, confirming that the space-group symmetry of the Ge crystal is preserved to the extent that the (0 0 4*n*+2) reflection remains symmetrically forbidden [Fig. 2 ▸(*c*)].

**Figure 3:**
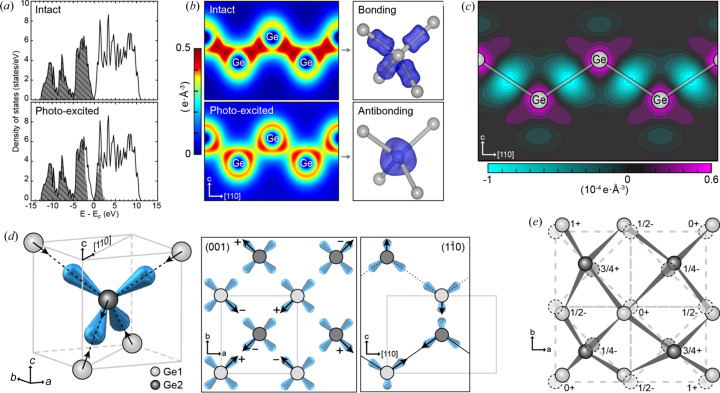
Photoinduced orbital reconfiguration and ionic displacements. (*a*) DFT calculation of electron density distributions for the intact and photoexcited states (10% of valence electrons upshift). Electron-filled states are shown by shaded gray regions. (*b*) Spatial charge distribution from DFT (see Section S4 the supporting information for details). Bonding electrons are photo-depleted to occupy anti-bonding orbitals weakening the interatomic bonding. (*c*) Changes in the charge distribution after photoexcitation are emphasized by subtracting the charge distribution of the intact state, which manifests the charge density reduction at the bonding site between Ge ions (green) and increases the anti-bonding sites (magenta). (*d*) Structure model with the photo-induced ionic displacements in a Ge tetrahedron. Ge ions are displaced along the [111] equivalent direction, breaking the local tetrahedral site symmetry. Ionic displacements are shown via the (001) and (110) plane viewing perspectives, displaying a Ge tetrahedron (gray box) within a conventional unit cell. (*e*) Atomic structure with the photoinduced ionic displacement with the lower site symmetry of 3*m* at the 32*e* position is shown to be consistent with the observed ATS reflection (see Section S3 of the supporting information for details).

We attributed this (0 0 6) intensity enhancement to the photoinduced changes in the empty density-of-states and local symmetry changes of resonant atoms (Fig. 3 ▸). Screening by photoexcited electrons can be expected to modify the energy band structure, within several hundred femtoseconds, leading to the intensity change but for a short duration; the long-lasting intensity enhancement (∼7 ps) excludes this screening as a main mechanism for the intensity change. We have further confirmed this photoinduced charge redistribution from the bonding to the antibonding orbital from the density functional theory (DFT) calculation [Fig. 3 ▸(*a*)]. For the DFT calculation, the electron population was changed by broadening the Fermi–Dirac (FD) distribution, reflecting the photon energy transfer and corresponding electron temperature increase (see Section S4 of the supporting information for details). Indeed, the IR photoexcitation led to significant changes in the charge density distribution to lose the original bonding character with redistribution of the bonding electrons to anti-bonding states, supporting our interpretation of the ATS scattering [Figs. 3 ▸(*b*) and 3 ▸(*c*)]. Although not dominant, we do not exclude other contributions including adiabatic shift and ultrafast redistribution of the FD distribution, gap narrowing, exciton, electron–hole plasma formation *etc.* (Shank *et al.*, 1983 ▸)

Rapid modification of the bonding electron configuration may also induce coherent ionic displacements [Figs. 3 ▸(*d*) and 3 ▸(*e*)]. This coherent displacement of the ions accommodates resonant scattering via the *E*1–*E*1 transition by lowering the original tetrahedral site symmetry of Ge ions. We have calculated the structure factor using a model with coherently displaced ionic positions to account for the (0 0 6) ATS and (0 0 4) Bragg reflections (see Section S3 of the supporting information for details). Our structure model that incorporates coherent displacements of Ge ions still preserves the overall crystal symmetry without disturbing the (0 0 4) Bragg reflection, but increases the intensity of the (0 0 6) ATS reflection consistent with the experimental results as described in Section S3 of the supporting information. Lattice thermalization can also drive random displacements of Ge ions incoherently, which can result in thermal-motion-induced ATS along with reduced Bragg reflection caused by the Debye-Waller effect (Kirfel *et al.*, 2002 ▸; Kokubun & Dmitrienko, 2012 ▸). However, this thermal contribution appears at a later delay time with enough energy transfer from the electron-to-phonon scattering, as shown by the decrease in the (0 0 4) Bragg reflection after a time lag of 7 ps at current laser fluence.

**Figure 4:**
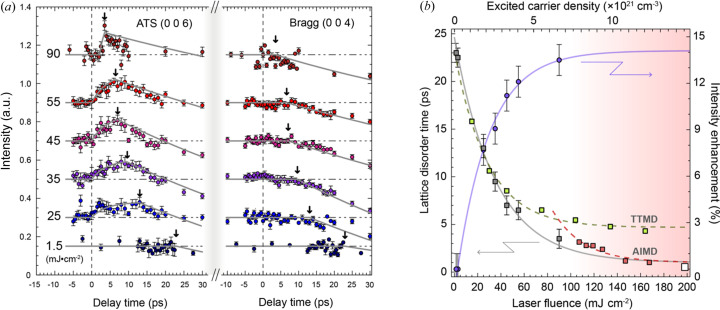
Laser fluence dependence of the lattice and orbital reflection. (*a*) Temporal evolution of the (0 0 6) orbital-sensitive and (0 0 4) Bragg intensities compared for different laser fluences. The onset time of lattice disorder becomes shorter for increased laser fluence (marked by the arrows). Solid lines are obtained from fits to single exponential functions (see Section S6 of the supporting information for details). (*b*) Fluence dependence of the intensity enhancement in the orbital-sensitive reflection and of the lattice disorder time is obtained. The excited carrier density was estimated considering the linear absorption process for reference (see Section S7 of the supporting information for details). The solid line is a guide to the eye. The experimental guide line (gray solid line) is extended to 200 mJ cm^−2^ by adapting reference data (open square point) reported in other work in a nonthermal melting regime (Sokolowski-Tinten *et al.*, 2001 ▸). Better consistency of the experimental results (gray square) with the TTMD (green squares and broken guideline) at low fluence and with the AIMD (red squares and broken guideline) at high fluence indicates the crossover from thermal-effect-dominated low-fluence kinetics to nonthermal lattice disorder at higher fluence. Error bars were obtained from the fits.

The laser-fluence-dependent investigation of the (0 0 6) orbital reflection intensity and the lattice disorder time, determined from the onset of the intensity change in the (0 0 4) Bragg reflection, provides further evidence on the tight correlation between bonding orbitals and lattice instability (Fig. 4 ▸). For the increased laser fluence, we observed two main features: faster onset time of the lattice disorder and stronger intensity of the orbital-sensitive (0 0 6) reflection. This indicates that the lattice disorder, in addition to the laser heating, is facilitated by disturbing the bonding orbitals.

In addition, we noted that the rates of change in both the lattice disorder time and the orbital intensity decreased by increasing the laser fluence [Fig. 4 ▸(*a*)]. This cannot be explained by a simple photo-absorption picture: 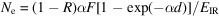 . Here, *F* denotes the laser fluence, *N*_e_ the number of photoexcited electrons, α the one-photon absorption, *d* the penetration depth, *E*_IR_ the IR photon energy of 1.54 eV and *R* the laser reflectance (Zürch *et al.*, 2017 ▸) (see Section S7 of the supporting information for details). In this simple relation, the photo-depletion of bonding electrons is expected to be amplified for a strong laser field with multiphoton absorption. Instead, we observed the opposite behavior displaying significantly reduced rates for laser fluences higher than ∼70 mJ cm^−2^, in both the orbital intensity enhancement and the lattice disorder time [Fig. 4 ▸(*b*)]. The lattice disorder time, varying from a few tens of picoseconds to sub-picosecond, showed a nonlinear dependence on the laser fluence to accompany ultrafast disorder with a saturated response in the orbital dynamics for laser fluences higher than ∼120 mJ cm^−2^. This sub-picosecond lattice disorder with saturated orbital reaction is consistent with the nonthermal melting of covalently-bonded materials (Stampfli & Bennemann, 1992 ▸; Sokolowski-Tinten *et al.*, 1998 ▸; Rousse *et al.*, 2001 ▸; Fritz *et al.*, 2007 ▸; Sciaini *et al.*, 2009 ▸).

To understand the fluence dependence, we performed a theoretical investigation using *ab initio* molecular dynamics (AIMD) and two-temperature molecular dynamics (TTMD) simulations (see Sections S8 and S9 the supporting information for details). The AIMD simulation was performed by broadening the FD distribution, reflecting photoexcited carrier densities consistent with laser fluences used in experiments. Ionic dynamics in AIMD are influenced exclusively by electron distributions to describe the electron-driven lattice disorder. In contrast, the TTMD reproduces ionic dynamics caused exclusively by increased kinetic energy from the scattering with electrons, *i.e.* the thermal reaction (Ihm *et al.*, 2019 ▸).

The lattice disorder time calculated from the two numerical simulations supported the experimental results at two different regimes, showing better consistency with the AIMD and TTMD results at higher and lower fluences, respectively [Fig. 4 ▸(*b*)]. The AIMD calculated for 1.2 eV broadening in the FD distribution, corresponding to the laser fluence of 170 mJ cm^−2^, showed sub-picosecond lattice disorder driven by charge depletion in the bonding orbitals and occupation in the antibonding orbitals [Fig. 4 ▸(*b*), see Section S9 of the supporting information for details]. The sub-picosecond saturation of the lattice disorder time obtained from the AIMD simulations reflected the nonthermal effects. Being rooted in the Born–Oppenheimer adiabatic approximation, the AIMD calculation does not consider the physical mechanism of electron–phonon energy transfer (Iftimie *et al.*, 2005 ▸). Instead, the ionic dynamics predicted from the AIMD simulations reflected the modification in interatomic PES due to the photo-induced electronic excitation. Photoinduced electron–hole plasma excitation disturbs the equilibrium ionic position by modifying the electronic PES, thereby inducing bond softening and displacive coherent phonon excitations to cause nonthermal melting (Hunsche *et al.*, 1995 ▸).

On the other hand, the lattice disorder time estimated from the TTMD simulation coincided well with the low-fluence experiment, being saturated at ∼5 ps on further fluence increase. This speed limit in inducing the lattice disorder is because the lattice thermalization requires sufficient time for the electron–phonon energy exchange as the essential photo-induced heating mechanism of TTMD. In comparison, bonding-electron-driven lattice disorder with the PES modification, as considered in the AIMD simulation, was a much faster process, surpassing such ionic thermalization speed limit. In the TTMD simulation, photoinduced modification of the electronic potential energy cannot be considered, and the atomic dynamics were driven by ionic kinetic energy influenced by the energy exchange from photo-excited hot electrons through electron–phonon scattering events. The rate of energy transfer from excited electrons to ions was determined by the electron–phonon coupling constant of Ge that also depends on the temperature to effectively expedite the lattice disorder time on the higher laser fluence. The saturating behavior of the lattice disorder time estimated from the TTMD calculation clearly illustrates that the speed of the ionic disorder becomes limited to several picoseconds, if only the thermal effect is considered.

Theoretical investigations have verified that our experimental observations with nonlinear fluence dependence resulted from the crossover behavior of the thermal–nonthermal kinetics on increasing the laser fluence. Interpreting photoinduced phase transition phenomena often starts by categorizing the dynamics involved as either thermal or nonthermal types without explicit criteria. Further, the bond softening due to depleted bonding electrons has been ascribed to drive photoinduced nonthermal lattice disorder but without direct evidence by monitoring the bonding orbitals. This study resolved these issues by investigating the interaction between the lattice and the 4*sp* ^3^ orbitals in Ge with the direct observation of the ultrafast bonding orbital dynamics and lattice disorder following the fs-IR laser illumination. The orbitals responded to the photoexcitation with local symmetry-changing coherent displacement of ions, whereas the lattice reacted with delay. The lattice disorder launched more rapidly by increasing the laser fluence which also accompanied a larger disturbance to the bonding orbitals as verified by fluence-dependent intensity variation in the (0 0 6) ATS reflection. On increasing the laser fluence to more than 70 mJ cm^−2^, the lattice disorder time showed a crossover behavior to approach sub-picosecond for fluences higher than 120 mJ cm^−2^; this occurred together with saturated intensity enhancement of orbital-sensitive reflections. The observation, also verified by the AIMD and TTMD simulations, showed continuous crossover reaction from more thermal kinetics toward ultrafast nonthermal kinetics for increased laser fluence.

The fluence-dependence results showed that electron reduction in the bonding orbital weakens the lattice stability, added to ionic thermalization, to facilitate the lattice disorder induced by photoexcitation. The impact of electron depletion in the bonding orbitals becomes stronger by increasing the laser fluence, which eventually disrupts the lattice exclusively by redistributing electrons from the bonding to the antibonding orbitals. By directly investigating the interplay between the bonding orbitals and lattice stability, we obtained a unifying picture, encompassing a thermal–nonthermal kinetic transition, behind the photo-induced lattice disorder, which is the essence of various ultrafast processes in nonequilibrium.

## 4. Summary

In this work, we performed time-resolved resonant X-ray scattering experiments at the PAL-XFEL to investigate the femtosecond dynamics of bonding orbitals related to the lattice order in the photo-induced ultrafast melting transition in a Ge crystal. Temporal evolution of the (0 0 6) ATS reflection with direct sensitivity to the bonding orbital is monitored to show that the photodepletion of bonding electrons leads to an immediate change in the orbital-sensitive reflection whilst the crystal lattice disorder launches with delayed response. The delayed response of the lattice disorder shows a nonlinear response on the pump laser fluence to vary from tens of picoseconds to a sub-picosecond delay. Theoretical investigation from *ab initio* and two-temperature molecular dynamics simulations corroborated the experimental observations at two different regimes to support thermal motion dominated the nonthermal melting picture for increased laser fluence. We verified that the photoexcitation of the electrons from the bonding orbital weakens the lattice stability to expedite the photo-induced lattice disorder, added to electron-lattice thermalization. This impact of bonding electron depletion becomes stronger by increasing the laser fluence, which eventually disrupts the lattice exclusively by redistributing electrons from bonding to antibonding orbitals. Our work provides a comprehensive understanding on the ultrafast melting process in nonequilibrium, explaining pump laser fluence-dependent crossover from thermal to nonthermal dominant dynamics.

## 5. Related literature

The following references are cited in the supporting information: Alavi *et al.* (1994 ▸); Henry & Straka (2010 ▸); Kameshima *et al.* (2014 ▸); Kresse & Furthmüller (1996 ▸); Mahdizadeh & Akhlamadi (2017 ▸); Norman *et al.* (2013 ▸); Perdew *et al.* (1996 ▸); Piaggi & Parrinello (2017 ▸); Plimpton (1995 ▸); Yin & Cohen (1982 ▸); Zijlstra *et al.* (2013 ▸).

## Supplementary Material

Supporting information figures and table. DOI: 10.1107/S2052252523007935/it5031sup1.pdf
