# Supplementary material for: Observing femtosecond orbital dynamics in ultrafast Ge melting with time-resolved resonant X-ray scattering
Source: IUCrJ. 2023 Sep 30;10(Pt 6):700–7. doi: 10.1107/S2052252523007935 (PMC10619452; doi:10.1107/S2052252523007935)
Supplement: Supplementary file 1 [file m-10-00700-sup1.pdf]

IUCrJ

Volume 10 (2023)

Supporting information for article:

Observing femtosecond orbital dynamics in ultrafast Ge melting with time-resolved resonant X-ray scattering

Heemin Lee, Je Young Ahn, Sae Hwan Chun, Do Hyung Cho, Daeho Sung, Chulho Jung, Jaeyong Shin, Junha Hwang, Sung Soo Ha, Hoyoung Jang, Byeong-Gwan Cho, Sunam Kim, Jaeku Park, Daewoong Nam, Intae Eom, Ji Hoon Shim, Do Young Noh, Yungok Ihm and Changyong Song

S1. Femtosecond time-resolved resonant X-ray scattering experiments

The tr-RXS experiments were performed using the X-ray scattering and spectroscopy beamline of the PAL-XFEL. Each single X-ray pulse was synchronized to a single fs-IR laser pulse (Ti-Sapphire laser with the pulse duration of ~ 100 fs and wavelength of 800 nm). The time-zero between the fs-IR laser and XFEL pulses at the sample position was defined by the inflection point of intensity reduction in thin Bi film (Kang *et al.*, 2017). The scattering experiments were performed in the horizontal scattering geometry with a dominant π-polarization for incident X-rays to collect ATS signals with π-to-σ and π-to-π channels simultaneously without resolving them. X-ray photon energy was tuned to Ge K-edge at 11.1095 keV using a double-crystal Si (111) monochromator. The angle between the optical axes of the incident XFEL and IR laser was maintained below ~ 5° to improve the temporal resolution. The XFEL beam was gently focused using stacks of Be compound refractive lenses to have a 100 μm diameter spot at the sample position. The beam size of the fs-IR laser was 500 μm in diameter, which is sufficiently large to ensure the spatial overlap between the XFEL and IR laser; this overlap was confirmed intermittently during the experiments using an inline optical microscope.

An undoped pure Ge bulk crystal of high-quality ($10\times10\times0.5$ mm^3) was selected to obtain RXS signals exclusively from electron orbitals. Epitaxially grown thin samples are often used for time-resolved experiments with matched pumping and probing volume of the samples. However, we intentionally avoided using this, as filmed samples are vulnerable to inducing lattice strain or defects from the film growth and generating refracted elastic waves from the interface, which are detrimental to the experiment by hampering the collection of signals exclusively from photo-excited electron orbitals. The mismatch between IR laser absorption length (~200 nm at λ = 800 nm) and X-ray extinction length (less than a micron) is present, but our interpretation of the experimental result on the onset of lattice disorder and ATS change is not influence by this volume mismatch. Under the specular scattering geometry, we can estimate the geometrical contribution of optical pump laser versus XFEL as $\eta_{HKL} \sim \frac{d_{pump\ laser}}{d_{XFEL}} = \frac{\sin(\theta_{HKL} + \Delta\theta_{pump\ laser})}{\sin(\theta_{HKL})}$, where $\Delta\theta_{pump\ laser}$ is angle difference between incident XFEL and pump laser ~ 10°. Because η_{004} (~1.39) is larger than η_{006} (~1.22), geometrical contribution of optical pump laser is larger for the (004) reflection than that of (006) reflection such that our interpretation of the disorder time estimated by changing (006) ATS reflection intensity and non-varying (004) Bragg reflection intensity is legitimate. Further investigation of temporal evolution of laser-heated volume is described in Supporting information 5.

S2. Single-pulse XFEL data collections

XRD data were collected using a dual-sensor multiport charge-coupled device detector (Kameshima *et al.*, 2014). XFEL pulses were delivered at 30 Hz, and data acquisitions were made by interleaving the IR laser illumination to allow data collection with alternating laser-on and laser-off conditions at a 15 Hz interval. XRD data collected for the IR laser-off condition verified the intactness of the crystal: full recovery of the crystalline lattice as well as the orbital-sensitive reflection to the initial intact condition. Any change in diffraction intensity in the laser-off condition indicates crystal damage; we avoided this by moving the illumination spot across the sample.

The diffraction intensity was obtained by integrating detector counts over reflection peak area above the noise levels. The obtained intensity was then normalized by the incident X-ray photon flux. A gas ionization monitor and a quadratic-beam-position monitor installed upstream of the X-ray focusing lens were used for flux calibration. The incident photon flux close to the sample interaction point was monitored by mounting Al foils in front of the sample and measuring the scattered intensity using a Si avalanche photodiode. Data collected from the laser-on condition was further normalized using the laser-off data obtained in the abovementioned alternating data collection scheme, which is essential to track photo-induced intensity variation accurately.

S3. Anisotropic X-ray tensor susceptibility in Ge and Photo-induced displacement of ions

The Ge crystal is a diamond structure with the space group symmetry of $Fd\bar{3}m$ (No. 227) and the Ge ions are at $8a$ symmetry positions, where the glide plane symmetry makes the (0 0 L=4*n*+2) reflections systematically forbidden. However, (0 0 4*n*+2) forbidden reflection can be observed when the anisotropic orbital sensitive X-ray form factor is considered, known as the anisotropic X-ray tensor susceptibility (ATS) (Dmitrienko *et al.*, 1983). This becomes realized in the RXS by tuning the X-ray energy to a specific core-shell binding energy of the atom, which promotes inter-atomic transitions between the corresponding core-shell to specific valence orbitals permitted by the selection rules.

The X-ray structure factor of the Ge for the (0 0 L) reflection is obtained as,

$$F_{Ge}(0\ 0\ \mathrm{L}) = \sum_{\{r_j\}} f_j exp\big(i2\pi \cdot \mathrm{L} \cdot z_j\big),$$

where $\{r_j\}$ sums over basis atoms in the unit cell and f_j denotes the corresponding atomic scattering factor of the j-th atom. The atomic scattering factor f is expressed by n-th order tensor as $f\ = f^0 + f_{res}\ = \ f^0 + f_{res}^{dd} + f_{res}^{dq} + \cdots$, where f^0 is the Thomson scattering term of scalar quantity. According to the second-order perturbation theory in nonmagnetic system, the tensorial component of resonant scattering factor f_{res} is given by,

$$f_{ab(res)} \sim \frac{\left\langle g_f \middle| (\boldsymbol{\varepsilon}_{\boldsymbol{a}}' \cdot \boldsymbol{p}) e^{-i\boldsymbol{k}' \cdot \boldsymbol{r}}, \middle| u \right\rangle \left\langle u \middle| (\boldsymbol{\varepsilon}_{\boldsymbol{b}} \cdot \boldsymbol{p}) e^{i\boldsymbol{k} \cdot \boldsymbol{r}}, \middle| g_i \right\rangle}{E_{\mathrm{bind}} - \hbar\omega - i\Gamma_u/2},$$

where $\boldsymbol{\varepsilon}$ and $\boldsymbol{k}$ indicate the polarization and the wave vector of incident X-rays, and $\boldsymbol{\varepsilon}'$ and $\boldsymbol{k}'$ represent those of scattered X-rays, respectively. As described in the main text, $g_{i(f)}$ describes the initial (final) core-level state ($g_f\ = g_i$ for elastic scattering in our work); u for the unoccupied valence shell with the energy bandwidth Γ_u, $E_{bind} = E_u - E_g$ for the core–shell binding energy, and $\hbar\omega$ for the energy of incident X-rays. By expanding the exponential factor, the resonant scattering factor can be expanded by dipole-dipole (*dd*, E1-E1), dipole-quadrupole (*dq*, E1-E2), and so on (Templeton *et al.*, 1994).

There are 8 Ge atoms cubic unit cell of the diamond structure with one set of Ge_1 at $\{r(x,y,z)\} = (0,0,0;\ ½,½,0;\ ½,0,½;\ 0,½,½)$ and the other of Ge_2 at (¼,¼,¼; ¾,¾,¼; ¾,¼,¾; ¼,¾,¾), being represented by two face-centered cubic crystals interleaved by (¼,¼,¼). Ge_1 ions and Ge_2 ions are related by an inversion center at the midpoint of the covalent bond and each set forms the tetrahedron around the other basis atom [Fig. 3(d)]. In this tetrahedral site symmetry $4\bar{3}m$, the ATS scattering from the E1–E1 transition is still forbidden, but the next higher-order terms, such as E1–E2, results nonzero resonant contribution to (0 0 4*n*+2) ATS reflection (Templeton *et al.*, 1994).

Here, we propose a structure model for the photoinduced displacements of the Ge atoms to occupy a lower symmetry site of $32e$, reduced from the initial $8a$ positions, preserving the original $Fd\bar{3}m$ space group of the crystal. A possibility to occupy other symmetry positions of $16c$ or $16d$ site is ruled out being incompatible with the symmetry lowering from ionic displacements. In the $32e$ positions $(3m)$, new coordinates of Ge ions are then $\{r(x,y,z)\} = (x,x,x;\ ½ - x, ½ - x, x;\ ½ - x, x, ½ - x;\ x, ½ - x, ½ - x)$ *for* Ge_1 , *and* $(¼ - x, ¼ - x, ¼ - x;\ ¾ + x, ¾ + x, ¼ - x;\ ¾ + x, ¼ - x, ¾ + x;\ ¼ - x, ¾ + x, ¾ + x)$ for Ge_2. With the deformation, displaced Ge atoms now occupy $32g$ symmetry position with the original site symmetry lowered to $3m$, which allows the E1-E1 transition as shown below.

The general form of tensorial scattering factor corresponding to the $3m$ site symmetry in dipole approximation after the similarity transformation is expressed as,

$$f_1 \sim \begin{bmatrix} a & b & b \\ b & a & b \\ b & b & a \end{bmatrix}$$

at site (x,x,x) and $(¼ - x, ¼ - x, ¼ - x)$, where a and b are phenomenological parameters. The tensorial atomic scattering factors of the other 6 Ge atoms are symmetrically related to by twofold rotations about the x, y and z axes as,

$$f_2 \sim \begin{bmatrix} a & b & -b \\ b & a & -b \\ -b & -b & a \end{bmatrix}$$

at site $(½ - x, ½ - x, x)$ and $(¾ + x, ¾ + x, ¼ - x)$,

$$f_3 \sim \begin{bmatrix} a & -b & b \\ -b & a & -b \\ b & -b & a \end{bmatrix}$$

at site $(½ - x, x, ½ - x)$ and $(¾ + x, ¼ - x, ¾ + x)$ and,

$$f_4 \sim \begin{bmatrix} a & -b & -b \\ -b & a & b \\ -b & b & a \end{bmatrix}$$

at site $(x, ½ - x, ½ - x)$ and $(¼ - x, ¾ + x, ¾ + x)$. With these, the tensorial structure factors for the (0 0 L) reflections for L=4n and 4n+2 are calculated as,

$$F_{Ge}(0\ 0\ 4n) \sim \cos\delta \begin{bmatrix} 8a & 0 & 0 \\ 0 & 8a & 0 \\ 0 & 0 & 8a \end{bmatrix} \sim \begin{bmatrix} 8a & 0 & 0 \\ 0 & 8a & 0 \\ 0 & 0 & 8a \end{bmatrix} \text{ and}$$

$$F_{Ge}(0\ 0\ 4n+2) \sim i\sin\delta \begin{bmatrix} 0 & 8b & 0 \\ 8b & 0 & 0 \\ 0 & 0 & 0 \end{bmatrix} \sim i\delta \begin{bmatrix} 0 & 8b & 0 \\ 8b & 0 & 0 \\ 0 & 0 & 0 \end{bmatrix},$$

where $\delta = 2\pi lx$ with small δ approximation. The structure factor of (0 0 4n) Bragg reflection does not contain polarization-switching off-diagonal terms nor any dependence on δ. Clearly, intensity of (0 0 4n+2) reflection increases by δ with the off-diagonal terms in the E1-E1 term. As a result, considering π-polarized incident XFEL pulses, the scalar form of structure factor is obtained as,

$$F_{Ge}(0\;0\;4n) \sim 8a\left[cos^2\left\{\frac{(2\theta)_{(0\;0\;4n)}}{2}\right\} - sin^2\left\{\frac{(2\theta)_{(0\;0\;4n)}}{2}\right\}\right] \text{ and}$$

$$F_{Ge}(0\;0\;4n+2) \sim 8bi\delta \cdot sin\left\{\frac{(2\theta)_{(0\;0\;4n+2)}}{2}\right\}.$$

As such, our structure model is consistent with the experimental results with constant (0 0 4) intensity and increasing (0 0 6) intensity with photo-induced coherent displacement before the thermal lattice disorder occurs. Relative magnitude of displacement can be extracted from the intensity enhancement of (0 0 6) ATS reflection [Fig. 4(b)] as displayed in Fig. S1. It should be noted that the optical creation of coherent phonon modes can induce such atomic displacements after femtosecond laser irradiation. The atomic displacements proposed in our structure model corresponds to optical phonon Γ_{25} mode (Yin & Cohen, 1982, Pfeifer *et al.,* 1992). For Ge atoms in the tetrahedral configuration, the anisotropic hole distribution can be transiently induced depending on the linear polarization direction of femtosecond laser pulse and the weakening of bonding also can be oriented preferentially, generating coherent lattice vibration (Pfeifer *et al.,* 1992).

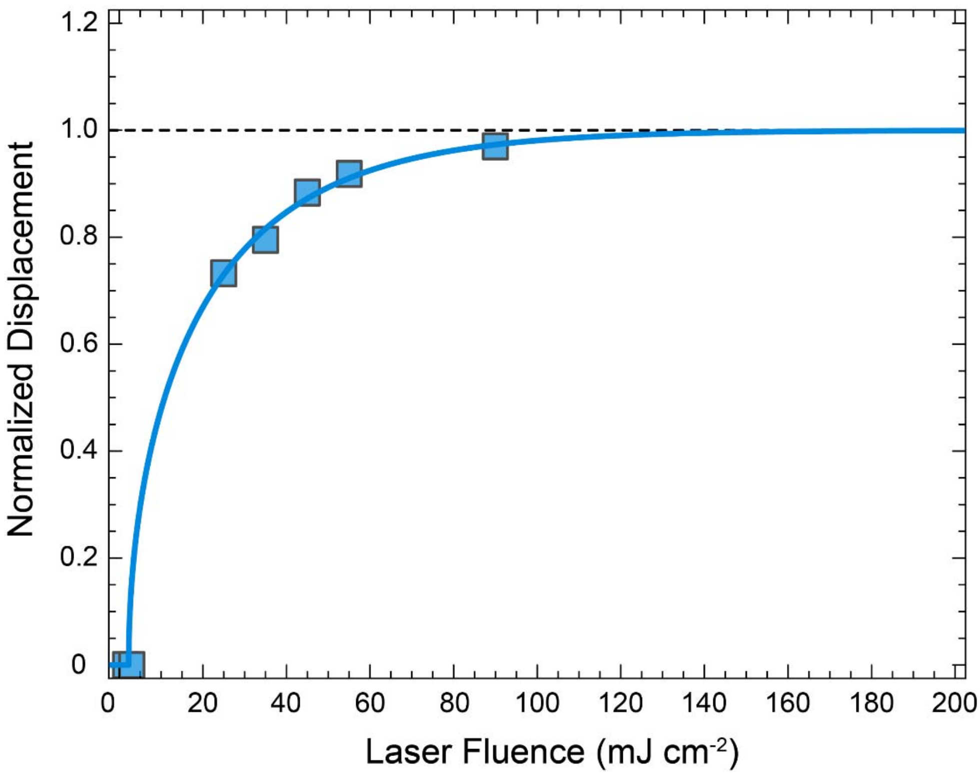

Figure S1 Estimated relative displacement of Ge ions using suggested structure model. Dashed line represents an asymptotic line of fitted single exponential function. Ignoring the displacement-

independent contributions (e.g. electron density part in resonant form factor), the magnitude of the saturated ionic displacement is roughly estimated as ~ 0.03 Å.

S4. Redistribution of charge density after the fs-IR laser excitation

Charge densities were studied for the FD broadening changing from 0.1 eV to 5.0 eV using the VASP code. As the width of broadening of electrons became larger from 0.1 eV in Fig. S2(a) to 5.0 eV in Fig. S2(c), the density of electron in bonding orbitals were diminished (Fig. S2). In the case of 5.0 eV broadening in Fig. S2(c), charge density contour was drawn showing further decreased electron density in the bonding orbitals. When about 10% of electrons was shifted up in Fig. S2, bonding orbitals almost disappear as shown in Fig. S2(d); the electrons in bonding orbitals decreased and moved to the anti-bonding orbitals.

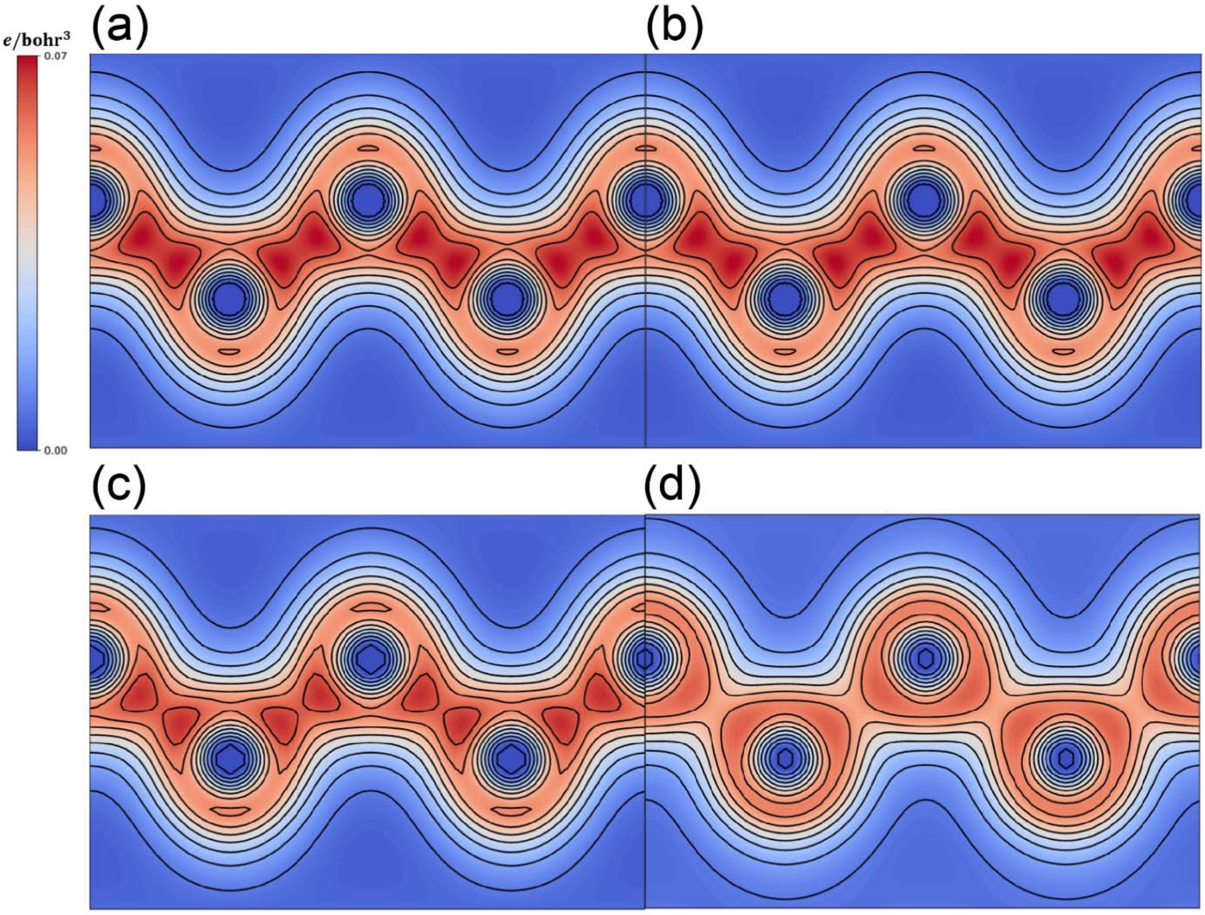

Figure S2 Charge density distribution in 4sp3 bonding and antibonding orbitals of Ge estimated for various electron broadening of 0.1 eV (a), 1.0 eV (b), 5.0 eV (c). (d) Charge distribution for the 10% electrons upshifted by approximately 1.0 eV above the other electrons.

S5. Thermal evolution of the Bragg reflection in bulk material

Here, we investigated temporal evolution of the heated volume to understand its influence on Bragg scattering intensity quantitatively. Development of thermalized ions was estimated using the decreased intensity of (0 0 4) Bragg reflection from the Debye-Waller effect. Heated portion of the scattering volume was modelled for two different situations to figure out more consistent situation with the experiment.

Model-I: Homogeneously heated volume over X-ray probe depth (~ 1 um) considers the ballistic motions with fermi velocity (~10 km/s) of the IR excited hot electrons to transfer excessive kinetic energy to ions within a several ps, time for electron and ion temperature equilibration from electron-phonon scattering. Here, the scattering intensity of the (0 0 4) Bragg reflection can be expressed as, $I_{DW} = I_0 exp\left(-\frac{1}{3}\langle dr^2\rangle G^2\right)$, where I_0 is the scattered intensity from the intact lattice assumed in room temperature, $\langle dr\rangle^2$ is mean-square-displacement (MSD) of the Ge ions, and G is reciprocal lattice vector that is $(0\ 0\ 4)\cdot 2\pi/a$ with lattice constant a in this case. Thermal MSD is estimated from the integrated intensity of the (0 0 4) Bragg intensity. The MSD follows the scaling relation as $\langle dr\rangle^2 \sim t^{\gamma}$, where the exponent γ characterizes the dynamical diffusion process of ions and t is the delay time (Zijlstra *et al.*, 2013). Intensity was fit to the relation as shown in Fig. S3(a), which resulted normal diffusive behavior from the beginning of disorder phase in blue solid line, with the exponent $\gamma = 0.96 \sim 1$, satisfying Fick's law of normal diffusion in isotropic liquid phase (Henry *et al.*, 2010). However, after about 200 ps, the exponent decreases to 0.45, indicating an anomalous diffusion. It may reflect increased scattering rate of ions, or possible inclusion of recrystallization at this time scale as well.

Model-II: In this model, we assumed the ions in the IR laser heated volume (*d* ~ 200 nm) were thermalized first from hot electrons, and temporal evolution of Bragg intensity is accounted for propagation of thermalized crystal with the volume fraction, α. In this case, diffraction intensity in kinematic diffraction can be obtained as a sum of X-ray scattering from hot and cold layers, $I = \alpha I_{DW} + \beta I_0\ (0 \leq \alpha \leq 1)$, where $\beta(t) = 1 - \alpha(t)$ is the volume of cold layer which is not thermalized yet. The MSD is obtained as hot layer proceeds into the bulk with the sound velocity of Ge ($5.5\ \mathrm{km \cdot s^{-1}}$) [Fig. S3(b)]. Here, the MSD shows clear fractional diffusion at about 60 ps, earlier than homogeneous thermalized case of model-I. As more volume is thermalized and it increases RMS and scattering rate of ions, resulting anomalous diffusive behavior. Single fractional diffusive motion can estimate time-resolved (0 0 4) intensity well [Fig. S3(c)].

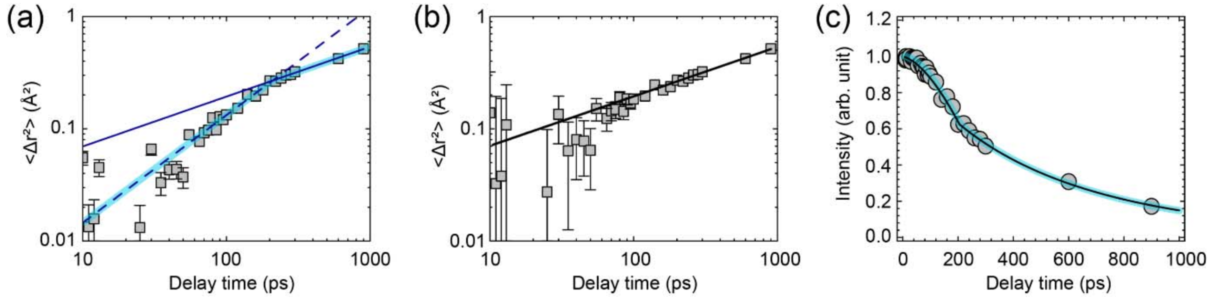

Figure S3 Intensity changes of Bragg reflection in bulk Germanium on lattice thermalization. (a) Time-dependent mean-square-displacements (MSD) considering only DW effect with nonequilibrium electrons from the (0 0 4) Bragg intensity. Normal diffusion is characterized in blue solid line and anomalous diffusion is drawn in blue broken line. (b) As ballistic motion of hot electrons is ignored, MSD shows different tendency with temporal evolving hot volume effect. Only anomalous diffusive behavior emerges in black solid line. (c) Diffraction intensity of (0 0 4) is fit for the MSD obtained for both models: Model-I in sky blue line and Model-II in black line.

S6. Fit functions used for time-resolved diffraction intensity

Time-resolved diffraction intensity of each reflections was fit to a single exponential,

$$y(t) = \mathrm{C}_1 \times \exp\left(-\frac{t - t_0}{\mathrm{C}_3}\right) + \mathrm{C}_2,$$

with t_0 the lattice-disorder time. Fit parameters of C_1, C_2 and C_3 are differently used depending on the reflection and temporal ranges shown in Table S1.

Table S1 Fitting parameters depending on different reflections and time domains.

	ATS (0 0 6), $0 < t < t_0$	ATS (0 0 6), $t > t_0$	Bragg (0 0 4), $t > t_0$
C_1	$-\frac{y(t_0) - 1}{\exp(t_0/\tau_1) - 1}$	A_2	A_3
C_2	$\frac{y(t_0)\exp(t_0/\tau_1) - 1}{\exp(t_0/\tau_1) - 1}$	$y(t_0) - \mathrm{A}_2$	$1 - \mathrm{A}_3$
C_3	τ_1	τ_2	τ_3

S7. Numerical values used for estimating excited carrier density

Number of excited carrier density was obtained using $N_e = (1 - R)\alpha\frac{F}{\hbar\nu}[1 - \exp(-\alpha d)]$, where F denoted the laser fluence, N_e for the number of photo-excited electrons, α for the one-photon absorption, d for the laser penetration depth, $\hbar\nu = E_{\mathrm{IR}}$ for the laser photon energy and R denoting laser reflectivity. In Fig. 4, we have used the following numerical values: α = 49322 cm^{-1}, R = 0.4221, d = 200 nm and $E_{\mathrm{IR}} = 1.54$ eV.

S8. Two-temperature molecular dynamics simulation

TTMD simulations were performed in a cell containing one million Ge atoms with dimensions of 28×28×28 nm3. A periodic boundary condition was imposed along directions perpendicular to the incident laser pulse. We used the Tersoff potential of Ge to describe interatomic interactions (Mahdizadeh *et al.*, 2017). The entire system was first equilibrated in canonical NVT ensemble at 300 K for 5 ps using LAMMPS. The TTMD simulations were then performed to estimate the thermal effects on Ge disorder, as implemented in LAMMPS (Norman *et al.*, 2013), in which the heat transfer from the electrons to the ions was evaluated using an inhomogeneous Langevin thermostat.

In the TTMD, the electron system is described at the continuum level using the relation described as follows: $C_e\rho_e\frac{\partial T_e}{\partial t} = \nabla\cdot(K_e\nabla T_e) - g_p(T_e - T_l) + \frac{F_{abs}}{\tau l_p}\exp(-x/l_{abs})$, where T_e, C_e, ρ_e and K_e are temperature, specific heat, density and thermal conductivity of the electron, and T_l and g_p describe ion temperature and electron-phonon coupling constant. Parameters of F_{abs}, τ and l_{abs} are used for describing the absorbed fluence, pulse duration, and the absorption length of the fs-IR laser, respectively. The electron specific heat was $C_e(T_e) = 0.02T_e\ \mathrm{mJ\cdot cm^{-3}\cdot K^{-1}}$ and the electron thermal conductivity was determined following $K_e(T_e) = D_e\cdot\rho_e\cdot C_e(T_e)$ with the thermal diffusion coefficient $D_e = 100\ \mathrm{cm^2\cdot s^{-1}}$ and the electron density $\rho_e = 2\times10^{13}\ \mathrm{cm^{-3}}$. The g_p of $2.7\times10^{10}\ \mathrm{W\cdot cm^{-3}}$, τ of 100 fs, l_p of 200 nm, and the laser absorption coefficient of 0.335 were used. The integration time step of 0.1 fs was used. The lattice-disorder time estimated from the TTMD calculations represents the time when the average lattice order parameter reaches $-2.708\ k_B$, which corresponds to ~ 900 K, after the fs-IR laser illumination.

S9. *Ab initio* molecular dynamics simulation

The disordering time of Ge in the nonthermal melting regime was determined using AIMD simulations. Our calculations were performed by the generalized gradient approximation and projector augmented wave method, as implemented in Vienna Ab initio Simulation Package (VASP) (Kresse *et al.*, 1996), wherein the forces on the ions are evaluated on-the-fly from the electronic structure obtained using DFT at adiabatic condition (Alavi *et al.*, 1994). In practice, the simulation cells containing 64, 256, 512, and 1728 atoms were used for the AIMD simulations. The PBE parameterization of the GGA for the exchange-correlated functionals (Perdew et al., 1996) was used and the energy convergence criterion is below 1.0×10^{-4} eV and the plane wave energy cutoff is 400 eV. The only one k-point Gamma-centered grid was used in all AIMD simulations. The simulation cells were equilibrated at 300 K in NVT (N: Number, V: Volume, T: Temperature) canonical ensemble for 4 ps. Nonthermal melting behavior was mimicked through high electronic broadenings from 0.8 eV ($T_e = 9{,}200$ K) to 1.2 eV ($T_e = 14{,}000$ K). These partial occupancies of electrons were determined by Fermi smearing, consistent with the carrier densities estimated from the experimental data [Fig. 4(b)], to simulate hot electron carriers. All AIMD simulations of the hot electron carriers were performed in the NVT ensemble at 300 K for 0.5 ps. From the AIMD results, the disordered times were calculated using four parameters about the disordered lattice entropy, which was implemented in the Large-scale Atomic/Molecular Massively Parallel Simulator (LAMMPS) code (Plimpton *et al.*, 1995, Piaggi *et al.*, 2017) [Fig. 4(b)].

Before the first procedures of calculation of the non-thermal melting picture, the crystalline Ge in the diamond structure that contains 256, 512, and 1728 atoms were equilibrated for 4 picoseconds by the AIMD that is implemented in VASP. After the equilibration, the system began with the broadenings in the Fermi-Dirac (FD) distribution to simulate the hot electron distributions right after the laser illumination at time zero. The partial occupancies are set by the Fermi smearing and set from 0.8 eV to 1.2 eV, which are equivalent from 6.32×10^{21} to 12.3×10^{21} $\text{electrons} \cdot \text{cm}^{-3}$ in the carrier density. Each carrier density was calculated by $\int_{E_C} g(E)f(E)dE$ where E_C is used for the energy of conduction band minimum, $g(E)$ for density of states, $f(E)$ for the FD distribution functions. The lattice entropy was calculated by the code which was implemented in LAMMPS.

Due to computational costs, we limited our studies such that the bigger systems containing 512 atoms and 1,728 atoms were considered not during entire time but during limited time for extracting lattice entropy parameters. The time profile of the lattice disorder showed the tendency known as a linear regression equation [Fig. S4(a)]. To describe the characteristics of the lattice disorder, four parameters were introduced for the lattice-disorder description. We defined the four parameters as A1 (starting), A2 (end), x_0 (time of slope p), and p (slope). By a linear extrapolation of the four parameters versus size of systems, the four parameters in an infinite system were obtained [Fig.

S4(b)]. From these parameters, the lattice disordered times that is the time when the lattice almost reached to the disordered state were estimated in each electron broadening case.

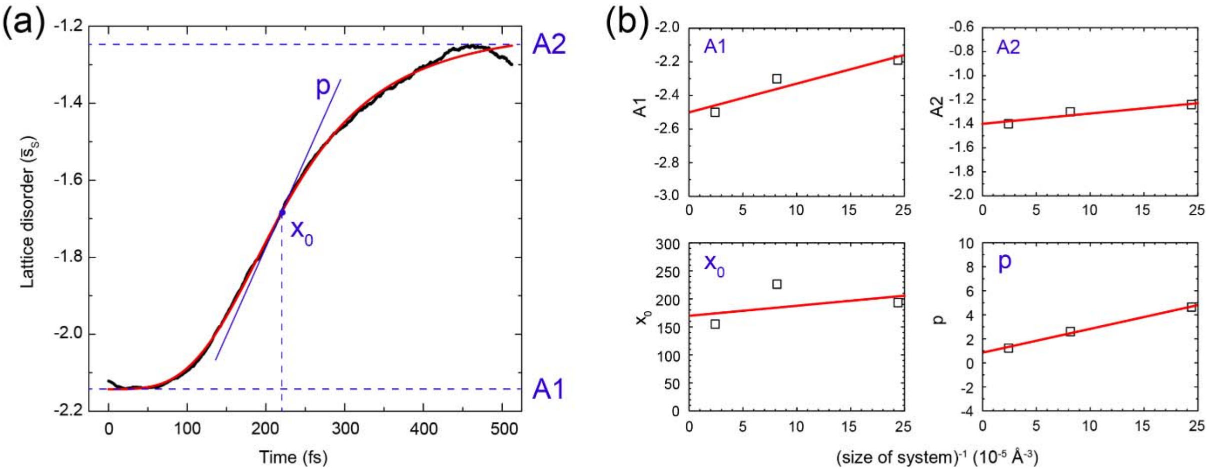

Figure S4 Analysis of the lattice entropy parameter A1, A2, x_0 and p. (a) The temporal evolution of the lattice disorder for the case of system size, 256 atoms, with 0.93 eV in the electron broadening. (b) The extrapolation of the inverse of size of system of the lattice entropy parameter A1, A2, x_0 and p.

SI Reference

Alavi, A., Kohanoff, J., Parrinello, M. & Frenkel, D. (1994). *Phys. Rev. Lett.* **73**, 2599-2602.

Dmitrienko, V. (1983). *Acta Crystallographica Section A: Foundations of Crystallography* **39**, 29-35.

Henry B. I., L. T. A. M., and Straka P. (2010). *Complex physical, biophysical and econophysical systems: World scientific lecture notes in complex systems.* Singapore: World Scientific.

Kameshima, T., Ono, S., Kudo, T., Ozaki, K., Kirihara, Y., Kobayashi, K., Inubushi, Y., Yabashi, M.,Horigome, T., Holland, A., Holland, K., Burt, D., Murao, H. & Hatsui, T. (2014). *Rev. Sci. Instrum.* **85**, 033110.

s

Kang, H.-S., Min, C.-K., Heo, H., Kim, C., Yang, H., Kim, G., Nam, I., Baek, S. Y., Choi, H.-J., Mun, G., Park, B. R., Suh, Y. J., Shin, D. C., Hu, J., Hong, J., Jung, S., Kim, S.-H., Kim, K., Na, D., Park, S. S., Park, Y. J., Han, J.-H., Jung, Y. G., Jeong, S. H., Lee, H. G., Lee, S., Lee, S., Lee, W.-W., Oh, B., Suh, H. S., Parc, Y. W., Park, S.-J., Kim, M. H., Jung, N.-S., Kim, Y.-C., Lee, M.-S., Lee, B.-H., Sung, C.-W., Mok, I.-S., Yang, J.-M., Lee, C.-S., Shin, H., Kim, J. H., Kim, Y., Lee, J. H., Park, S.-Y., Kim, J., Park, J., Eom, I., Rah, S., Kim, S., Nam, K. H., Park, J., Park, J., Kim, S., Kwon, S., Park, S. H., Kim, K. S., Hyun, H., Kim, S. N., Kim, S., Hwang, S.-m., Kim, M. J., Lim, C.-y., Yu, C.-J., Kim, B.-S., Kang, T.-H., Kim, K.-W., Kim, S.-H., Lee, H.-S., Lee, H.-S., Park, K.-H., Koo, T.-Y., Kim, D.-E. & Ko, I. S. (2017). *Nat. Photon.* **11**, 708-713.

Kresse, G. & Furthmüller, J. (1996). *Phys. Rev. B* **54**, 11169-11186.

Mahdizadeh, S. J. & Akhlamadi, G. (2017). *J. Mol. Graph. Model.* **72**, 1-5.

Norman, G. E., Starikov, S. V., Stegailov, V. V., Saitov, I. M. & Zhilyaev, P. A. (2013). *Contrib. Plasm. Phys.* **53**, 129-139.

Perdew, J. P., Burke, K & Ernzerhof, M. (1996). *Phys. Rev. Lett* **77**, 3865.

Pfeifer, T., Kütt W., Kurz H. and Scholz R. (1992). *Phys. Rev. Lett* **69**, 3248.

Piaggi, P. M. & Parrinello, M. (2017). *J. Chem. Phys.* **147**, 114112.

Plimpton, S. (1995). *J. Comp. Phys.* **117**, 1-19.

Templeton, D. H. & Templeton, L. K. (1994). *Phys. Rev. B* **49**, 14850-14853.

Yin, M. T. & Cohen, M.L. (1982). *Phys. Rev. B* **26**, 3259.

Zijlstra, E. S., Kalitsov, A., Zier, T. & Garcia, M. E. (2013). *Adv Mater.* **25**, 5605-5608.
